# Influence of the Stability of a Fused Protein and Its Distance to the Amyloidogenic Segment on Fibril Formation

**DOI:** 10.1371/journal.pone.0015436

**Published:** 2010-11-23

**Authors:** Anja Buttstedt, Reno Winter, Mirko Sackewitz, Gerd Hause, Franz-Xaver Schmid, Elisabeth Schwarz

**Affiliations:** 1 Institute for Biochemistry and Biotechnology, Martin-Luther-University Halle-Wittenberg, Halle, Germany; 2 Biocenter of the Martin-Luther-University Halle-Wittenberg, Halle, Germany; 3 Biochemical Laboratory, University of Bayreuth, Bayreuth, Germany; University of South Florida College of Medicine, United States of America

## Abstract

Conversion of native proteins into amyloid fibrils is irreversible and therefore it is difficult to study the interdependence of conformational stability and fibrillation by thermodynamic analyses. Here we approached this problem by fusing amyloidogenic poly-alanine segments derived from the N-terminal domain of the nuclear poly (A) binding protein PABPN1 with a well studied, reversibly unfolding protein, CspB from *Bacillus subtilis*. Earlier studies had indicated that CspB could maintain its folded structure in fibrils, when it was separated from the amyloidogenic segment by a long linker. When CspB is directly fused with the amyloidogenic segment, it unfolds because its N-terminal chain region becomes integrated into the fibrillar core, as shown by protease mapping experiments. Spacers of either 3 or 16 residues between CspB and the amyloidogenic segment were not sufficient to prevent this loss of CspB structure. Since the low thermodynamic stability of CspB (Δ*G*_D_ = 12.4 kJ/mol) might be responsible for unfolding and integration of CspB into fibrils, fusions with a CspB mutant with enhanced thermodynamic stability (Δ*G*_D_ = 26.9 kJ/mol) were studied. This strongly stabilized CspB remained folded and prevented fibril formation in all fusions. Our data show that the conformational stability of a linked, independently structured protein domain can control fibril formation.

## Introduction

Congenital protein misfolding diseases moved into the focus of intensive investigation during the past decades (for review see [1]). In addition to point mutations as in the cases of familial Alzheimer's or Parkinson's Disease [2], [3], trinucleotide expansions can also cause protein misfolding diseases [4]. The best-known example for disease-causing trinucleotide expansions is Chorea Huntington, a disease provoked by extensive expansions of CAG codons in the first exon of the gene for huntingtin resulting in stretches of up to 200 Gln residues [5]. Besides expansions of Gln codons, extensions of Ala segments occur as well and can lead to diseases and/or developmental disorders [6], [7].

**Figure 1:**
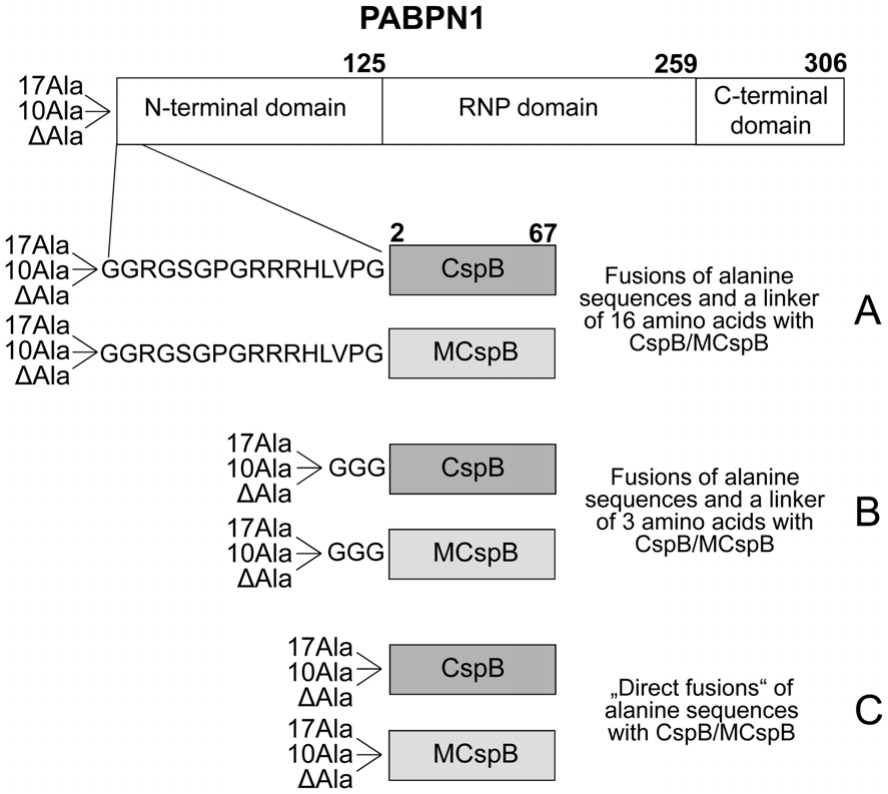
Schematic representation of the fusions analyzed in this work (modified according to [**19**]). For all linkers and CspB mutants, three N-terminal fusions were produced: 17Ala corresponds to the most extreme alanine extension observed in man, 10Ala presents the wild type sequence and ΔAla is a negative control. The alanine peptides are followed by the tripeptide GAA.

The disease oculopharyngeal muscular dystrophy (OPMD) is histochemically characterized by amyloid-like intranuclear inclusions consisting mainly of the nuclear RNA binding protein PABPN1 [8]–[11]. This protein regulates the length of poly(A) mRNA tails [12], [13]. The wild type protein contains a segment of ten consecutive Ala residues in its N-terminal domain (Fig. 1). In OPMD patients, extensions of this segment by up to seven additional Ala residues have been found [14].

The recombinantly produced wild type form of the N-terminal domain of PABPN1 with ten Ala residues forms fibrils very slowly [15] but faster when this poly-Ala stretch was extended to 17 residues. The fibrils were exceptionally stable; they could not be completely solubilized and unfolded by strong chemical denaturants [16]. Therefore their thermodynamic stability cannot be measured. Here we addressed the interrelation between protein stability and fibril formation indirectly by fusing a protein with a well characterized thermodynamic stability, the cold shock protein B (CspB) from *Bacillus subtilis* [17] with amyloidogenic oligo-Ala segments of PABPN1. CspB unfolds and refolds extremely fast [18], and therefore it is a good model protein for analyzing thermodynamic parameters affecting fibril formation. Previous results had shown that the CspB moiety remains folded in fibrils formed by fusions between the entire N-terminal domain of PABPN1 and CspB [19]. In contrast, CspB was forced to unfold during fibril formation when the amyloidogenic oligo-Ala segment was fused directly with CspB [19].

In this study, we explored how the folded state of CspB in the fibrils depends on the length of the linker between the amyloidogenic oligo-Ala segment and CspB. In a reciprocal approach, we investigated how fibril formation is affected when the amyloidogenic segment is linked with a thermodynamically stabilized variant of CspB [20]. Our results indicate that CspB folding and fibril formation are mutually exclusive even when CspB and the oligo-alanine segment are separated by 16 residues. In a reciprocal fashion, fibril formation is impaired when the amyloidogenic segment is fused to a stabilized CspB variant, indicating that stabilization of an adjacent folded domain inhibits fibril formation.

## Materials and Methods

### Recombinant constructs, protein expression and purification

For production of variants with linkers of 3 or 16 amino acids, a fusion construct consisting of the N-terminal domain of PABPN1 and CspB served as a template [19]. The linker variants A and B were constructed by deletion of the respective nucleotide sequence for *PABPN1* using the QuickChange II Site-Directed Mutagenesis Kit (Stratagene) and the following primers: 5′-GCA GGA GCA GCA GGA GGA GGC TTA GAA GGT AAA GTA AAA TGG-3′ for the 10Ala and 17Ala constructs of generic construct B, 5′-AGC CAT ATG GCA GGA GGA GGC TTA GAA GGT AAA GTA AAA TGG-3′ for the deletion (ΔAla) variant of construct B and 5′-GAA GAC ATT TAG TAC CAG GAT TAG AAG GTA AAG TAA AAT GG-3′ for the generic construct A (16 residue linker). The reverse primers had the reverse complementary sequences. PCR products were treated with *Dpn*I and transformed in *E. coli* XL1-Blue. Similarly, the A46K/S48R mutation was introduced into the coding DNA for CspB via the QuickChange II Site-Directed Mutagenesis Kit. For this mutagenesis, the primer 5′-CTT TAG AAG AAG GCC AAA AAG TTC GTT TTG AAA TCG TTG AAG G-3′ and its reverse complement were used. Recombinant gene expression and protein purification was performed according to [19] except that heat precipitation was omitted.

### Determination of the protein concentration

Protein purity and concentrations of the soluble species were assessed by UV spectroscopy recording readings at 280 nm. The molar extinction coefficients were determined as 5500 M^−1^ cm^−1^ for generic constructs A, B and C [21]. Protein concentrations of fibrils were calculated by determination of the amount of the single tryptophan residue under denaturing conditions as published [19], [22].

### Proteolysis

Proteolysis of the fibrils was performed with proteinase K. Fibrils were harvested by centrifugation for 1 h at 260 000 *g* in an Optima TM TLX ultracentrifuge, washed with 2 M (10Ala-CspB) or 1.5 M (17Ala-L3/L16-CspB) guanidinium chloride (GdmCl), 5 mM KH_2_PO_4_, 100 mM NaCl, pH 7.5 and again separated by centrifugation. Fibrils were resuspended in 50 mM Tris/HCl, 100 mM NaCl, 2 mM CaCl_2_, pH 8.0, and the protein concentration was adjusted to 1 to 5 mg/ml. Proteinase K was added at a ratio of 1∶50 (w/w), and the solution was incubated for 2 h at 37°C. Reactions were stopped by adding PMSF. After centrifugation for 1 h at 260 000 *g*, the pellet was first washed with 2 M or 1.5 M GdmCl, 5 mM KH_2_PO_4_, 100 mM NaCl, pH 7.5, respectively and then twice with 5 mM KH_2_PO_4_, 100 mM NaCl, pH 7.5. Fibrils of 10Ala-CspB were solubilized with 5 M guanidinium thiocyanate (GdmSCN), 0.1% trifluoroacetic acid (TFA) (v/v) over night at room temperature. The solution was again centrifuged, and the supernatant analyzed by reversed phase HPLC. Peptides were separated on a Nucleosil 5u C18 (125×4 mm 5 micron) column (Phenomenex, Aschaffenburg, Germany) at a flow rate of 0.7 ml min^−1^. After injection of the solution, the column was flushed with H_2_O, 0.05% TFA (v/v) for 3 min. Peptides were eluted with an increasing gradient of acetonitril (AcN), 0.05% TFA (v/v) (0–50% in 70 min and 50–100% in 17 min). The separated peptides were analyzed by MALDI-TOF mass spectrometry using a Reflex II-mass spectrometer (Bruker Daltonik GmbH, Germany). For the alignment of the proteolytic fragments, the program FindPept (http://www.expasy.ch/tools/findpept.html) was used. Fibrils of 17Ala-L3/L16-CspB were solubilized with 6 M GdmCl, 5 mM KH_2_PO_4_, 100 mM NaCl, pH 7.5 and analyzed by fluorescence spectroscopy in the same buffer.

### Circular dichroism (CD) and fluorescence spectroscopy

CD spectra were recorded with a Jasco J810 spectropolarimeter in 5 mM KH_2_PO_4_, 100 mM NaCl, pH 7.5 at 20°C at protein concentrations of 0.85 to 1.1 mg/ml in 0.1 mm cuvettes. The spectra were measured 10 times and averaged. Data were collected in 0.1 nm steps. Spectra were buffer corrected. Tryptophan fluorescence was recorded with a Jobin Yvon Fluoromax2 equipped with a thermostatted cell holder. Measurements were performed in 5 mM KH_2_PO_4_, 100 mM NaCl, pH 7.5 at 20°C at a protein concentration of 3 µM. The excitation wavelength was 295 nm at a bandwidth of 5 nm. Spectra were recorded in 1 cm cuvettes at a bandwidth of 5 nm and accumulated three times. Data were averaged and buffer corrected.

### Unfolding and refolding transitions

Thermal unfolding transitions were measured in 5 mM KH_2_PO_4_, 100 mM NaCl, pH 7.5, at protein concentrations of 0.15 to 0.22 mg/ml in 0.1 cm cuvettes. The transitions were monitored by changes of the CD signals at 205 nm at 1 nm band width. Heating rates were 1°C min^−1^. Transitions were evaluated using a non-linear least-square fit according to a two state model [23]. In this procedure, a constant value of 4 kJ mol^−1^ K^−1^ was used for the change in heat capacity upon unfolding (ΔC_P_). For urea-induced unfolding, urea (Ultrapure grade from USB, Ohio, USA) concentrations were determined by refraction [24]. Proteins were diluted to concentrations of 3 µM in 5 mM KH_2_PO_4_, 100 mM NaCl, pH 7.5 containing various urea concentrations. Denaturation was allowed to proceed for 1 h at 20°C. Fluorescence was recorded at an emission wavelength of 345 nm upon excitation at 295 nm with a Jobin Yvon Fluoromax2 at 20°C. Experiments were performed in 1 cm cuvettes with excitation and emission band width of 5 nm each. The unfolding transitions were analyzed by assuming a two-state transition between the folded (N) and unfolded (U) conformation. For obtaining the thermodynamic parameters Δ*G*_D_ and *m* values, non-linear least-square fits were performed [25].

### dT7 binding assay

The single-stranded DNA fragment dT7 5′-TTTTTTT-3′ was purchased from Thermo Electron GmbH (Germany). Concentrations of dT7 were calculated by the absorbance at 260 nm. The extinction coefficient of dT7 at 260 nm was determined as 58 800 M^−1^ cm^−1^ according to [26]. Binding was measured by fluorescence as described [19].

### Fibril analysis

Proteins were dissolved to final concentrations of 0.5 mM and incubated at 37°C. The buffer was 5 mM KH_2_PO_4_, 100 mM NaCl, pH 7.5 containing 1 µg/ml NaN_3_. Seeded reactions contained 5% (w/w) seeds prepared from fibrils of 10Ala-CspB. Seeds were prepared as described previously [16] and added immediately after preparation. For determining ANS or ThT fluorescence, samples were briefly mixed, and aliquots were diluted into 50 µM ANS or 5 µM ThT in 5 mM KH_2_PO_4_, 100 mM NaCl, pH 7.5 at final concentrations of 5 µM. Fluorescence was recorded with a Jobin Yvon Fluoromax2 upon excitation at 370 nm and at an emission wavelength of 480 nm at 20°C. Experiments were performed in 1 cm cuvettes with an excitation and emission slit width of 5 nm. For EM analysis, carbonized copper grids (Plano, Wetzlar, Germany) were used, prepared as published [16], and visualized in a Zeiss EM 900 electron microscope operating at 80 kV.

## Results

### Protease mapping of fibrils formed by fusions between the amyloidogenic segment and CspB

**Figure 2:**
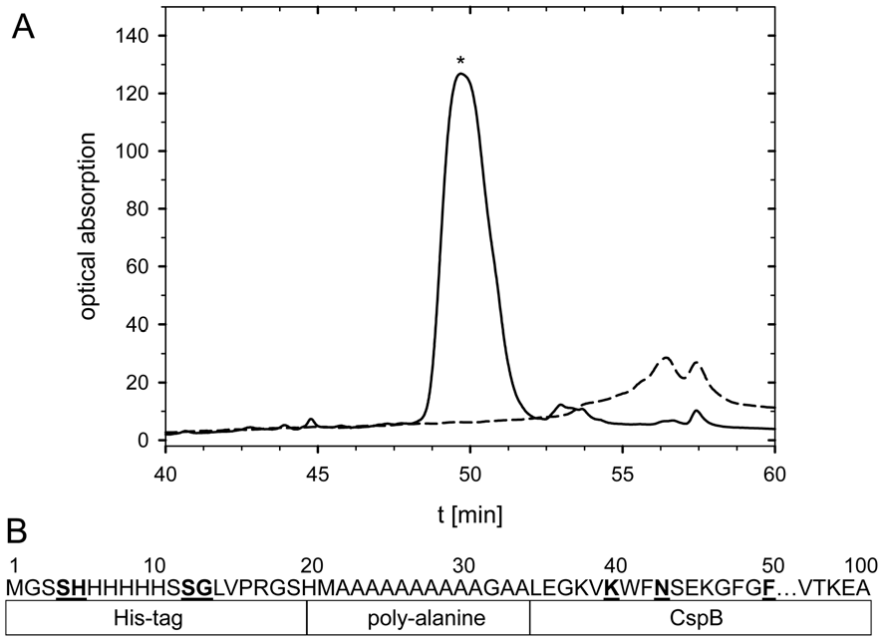
Determination of the fibrillar core of the “direct fusions”. **A** RP-HPLC chromatograms of intact and protease digested solubilized fibrils of 10Ala-CspB. Intact fibrils, dashed line; proteinase K digested fibrils, solid line. The chromatograms were recorded by UV absorption at a wavelength of 210 nm. The peaks were collected and analyzed by mass spectrometry. **B** Schematic representation of the proteolysis protected amino acid sequence of the fibrils of 10Ala-CspB. The underlined and bold amino acids represent the beginning and the end of the protected segments.

In previous work we had shown that when CspB is fused with the complete N-terminal domain of PABPN1 fibrils are formed in which the CspB moiety remained folded. In contrast, in fibrils arising from “direct fusions” between the oligo-Ala stretch and CspB (fusions C, Fig. 1), the CspB moiety was unfolded, presumably because part of it was recruited to the fibrils [19]. To determine to what extent CspB becomes incorporated into the protease-resistant core of the fibrils, fibrils from 10Ala-CspB (Fig. 1C) were treated with proteinase K. Protease-resistant fibrillar material was recovered by centrifugation. After solubilization with 5 M guanidinium thiocyanate, the peptide fragments were separated by RP-HPLC (Fig. 2A) and identified by mass spectrometry. The dominant peak (marked by an asterisk in Fig. 2) represents peptides that extend until Lys7, Asn10, or Phe17 of CspB (supporting information, Table S1) (Fig. 2B). Similar results were obtained with thermolysin digestion (data not shown). These results indicate that 5-15 N-terminal residues of CspB became integrated into the fibrils and were thus protected from proteolysis. This explains why fibril formation leads to unfolding of CspB when it is linked directly with the oligo-Ala stretch. A similar protease mapping experiment with the N-terminal domain of PABPN1 possessing the OPMD-associated extension of seven additional alanines had shown that in this case the fibrillar core structure also extended by several residues beyond the oligo-Ala segment [27].

### Stability of fusion proteins with linkers of 3 and 16 residues between the amyloidogenic segment and CspB

To explore how the coupling between fibrillation and unfolding of CspB depends on the length of the linker between the oligo-Ala stretch and CspB, we constructed several fusion proteins in which the length of the amyloidogenic peptide (10 or 17 Ala residues) and the length of the linker (3 or 16 residues) were varied. In addition, control proteins were produced, in which only the 3- or 16-residues linkers, but not the oligo-alanine stretches were fused to CspB. In the nomenclature of the variants, “10Ala” and “17Ala” denote the length of the amyloidogenic alanine stretch, “L3” and “L16” denote the length of the linker to the CspB moiety (Fig. 1). These linkers, including the GAA tripeptide, correspond to the sequence of the N-terminal domain that follows the Ala stretch in wild type PABPN1.

**Figure 3:**
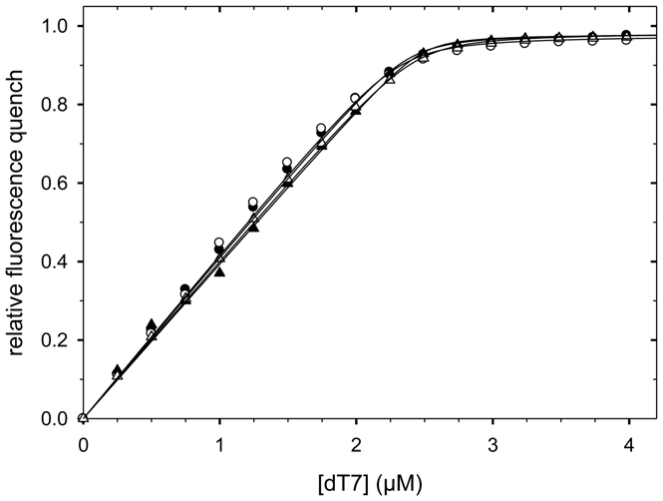
Nucleic acid binding of CspB. Binding of dT7 to the fusion proteins: Variants with 10 alanines are shown with filled symbols; variants with 17 alanines with open symbols; variants with a linker of 3 amino acids are shown as circles; variants with a linker of 16 amino acids as triangles. The corresponding *K*_D_ values are listed in Table 1. Measurements were carried out in 5 mM KH_2_PO_4_, 100 mM NaCl, pH 7.5 at 20°C.

The recombinantly produced variants were purified to homogeneity (supporting information, Fig. S1), and their stabilities and functions were determined. In both cases, Trp8 of CspB could be used as a sensitive reporter residue. The functionality of the CspB moieties in the fusion proteins was assayed by their ability to bind with high affinity to an oligonucleotide consisting of seven deoxy-thymidines (dT7) [28], [29]. This binding strongly quenches the fluorescence of Trp8 of CspB, which resides at the oligonucleotide binding site [30]–[33]. Fig. 3 shows four representative titration curves, Table 1 lists the *K*_D_ values for all variants. They show that the high affinity of CspB for the oligonucleotide dT7 is retained in all fusion proteins. Thus, CspB was correctly folded and functional (Table 1).

**Table 1:** *K*_D_ values upon dT7 binding and thermodynamic parameters of CspB.

Protein	*K*_D_ (nM)	Δ*G*_D_ (kJ/mol)	*m* (kJ/[mol M])	T_M_ (°C)
CspB	23.4±5.2*	12.4±0.9*	3.7±0.3*	52.1±0.1
10Ala-CspB	24.5±2.3*	6.0±0.5*	3.0±0.2*	41.5±0.1
17Ala-CspB	16.5±3.6*	5.9±0.3*	3.3±0.1*	40.3±0.5
ΔAla-L3-CspB	15.4±4.3	9.8±0.6	4.3±0.2	40.0±0.1
10Ala-L3-CspB	14.8±3.7	6.0±0.5	3.5±0.2	36.7±0.3
17Ala-L3-CspB	20.7±4.8	6.0±0.4	4.0±0.2	35.6±0.2
ΔAla-L16-CspB	12.8±2.3	6.8±0.4	3.2±0.1	40.3±0.4
10Ala-L16-CspB	8.2±2.8	7.8±0.6	3.4±0.2	40.6±0.3
17Ala-L16-CspB	17.9±2.0	7.9±0.5	3.6±0.2	40.3±0.3
MCspB (A46K/S48R)	23.8±2.8	26.9±2.0	4.8±0.4	67.2±0.1
10Ala-MCspB	17.7±1.0	16.6±0.6	3.5±0.1	60.2±0.1
17Ala-MCspB	18.7±1.1	16.6±1.1	3.6±0.2	61.5±0.1
ΔAla-L3-MCspB	15.0±2.9	15.1±0.6	3.4±0.1	60.3±0.1
10Ala-L3-MCspB	13.2±7.3	14.5±0.6	3.4±0.1	58.6±0.1
17Ala-L3-MCspB	18.2±4.7	12.8±0.6	3.2±0.1	58.4±0.1
ΔAla-L16-MCspB	12.6±4.4	17.5±0.7	3.8±0.1	59.5±0.1
10Ala-L16-MCspB	12.3±3.8	15.1±0.7	3.3±0.1	59.3±0.1
17Ala-L16-MCspB	14.9±4.5	16.4±0.7	3.6±0.1	59.7±0.1

Parameter were derived from urea- and thermal-induced unfolding and refolding experiments. For comparison also the parameters for the negative controls, the ΔAla-variants, are shown. All measurements were carried out in 5 mM KH_2_PO_4_, 100 mM NaCl, pH 7.5 at 20°C. Measurements for the reference values indicated by

*were performed at 25°C [19].

**Figure 4:**
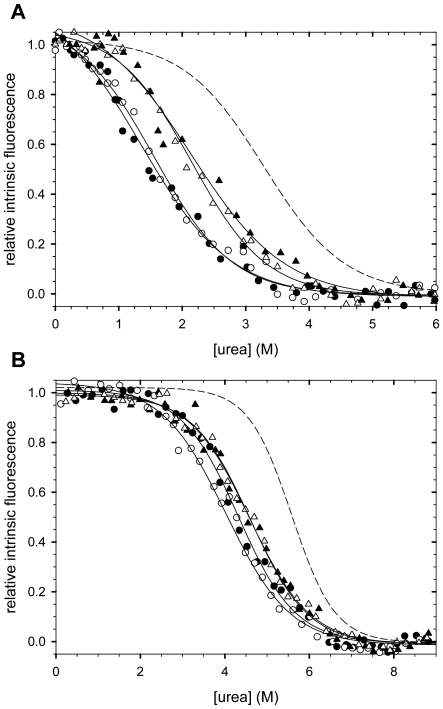
Urea-induced unfolding transitions of the fusions with CspB and MCspB. Variants with 10 alanines are shown with filled symbols; variants with 17 alanines with open symbols; variants with a linker of 3 amino acids are shown as circles; variants with a linker of 16 amino acids as triangles. The dashed line corresponds to the unfolding transition of unlinked CspB/MCspB. The corresponding Δ*G*_D_ and *m* values are indicated in Table 1. Measurements were carried out in 5 mM KH_2_PO_4_, 100 mM NaCl, pH 7.5 at 20°C. The full reversibility of the transitions is demonstrated in Fig. 2 of the supporting information. **A** Transitions of the Fusions with CspB, **B** Transitions of the Fusions with MCspB.

Subsequently, the thermodynamic stabilities of the CspB moieties of the fusion proteins were determined by measuring their urea-induced unfolding transitions. Unfolding leads to a decrease in fluorescence of Trp8 of CspB and thus provides a sensitive probe for its stability. Unfolding of all variants was fully reversible, and identical curves were obtained in refolding experiments (supporting information, Fig. S2A). Linker sequence and/or linker length exerted a strong influence on the stability of the CspB moiety in the fusion proteins (Fig. 4A). Isolated CspB showed a stability (Gibbs free energy of denaturation, Δ*G*_D_) of 12.4 kJ/mol under the conditions of our stability measurements [19]. When CspB was linked with 10 or 17 Ala residues via the short L3 linker (in the L3-variants), Δ*G*_D_ was reduced to 6.0 kJ/mol (Table 1). When the linker was extended to 16 residues (in the L16-variants), a slightly higher Δ*G*_D_ value of 7.8 kJ/mol was determined. Thermal unfolding transitions were recorded for all constructs as well. The rank order of the transition midpoints (Table 1) correlates well with the rank order of the Δ*G*_D_ values from the urea-induced unfolding transitions. Together, these thermodynamic data show that the stability of CspB is decreased in all fusion proteins. The strongest destabilization is observed when the CspB moiety is separated from the oligo-Ala stretch by only three residues. When only the L3 linker, but not the oligo-Ala stretch, is present, CspB shows a Δ*G*_D_ value of 9.8 kJ/mol (Table 1), which indicates that most of the destabilization observed for 10Ala- and 17Ala-CspB (approximately 4 kJ/mol relative to isolated CspB, Table 1) originates from the oligo-Ala moiety. Possibly, this segment interacts unfavourably with CspB in the fusion proteins.

### A linker of 16 residues between the amyloidogenic segment and CspB is not sufficient to maintain CspB folded in fibrils

**Figure 5:**
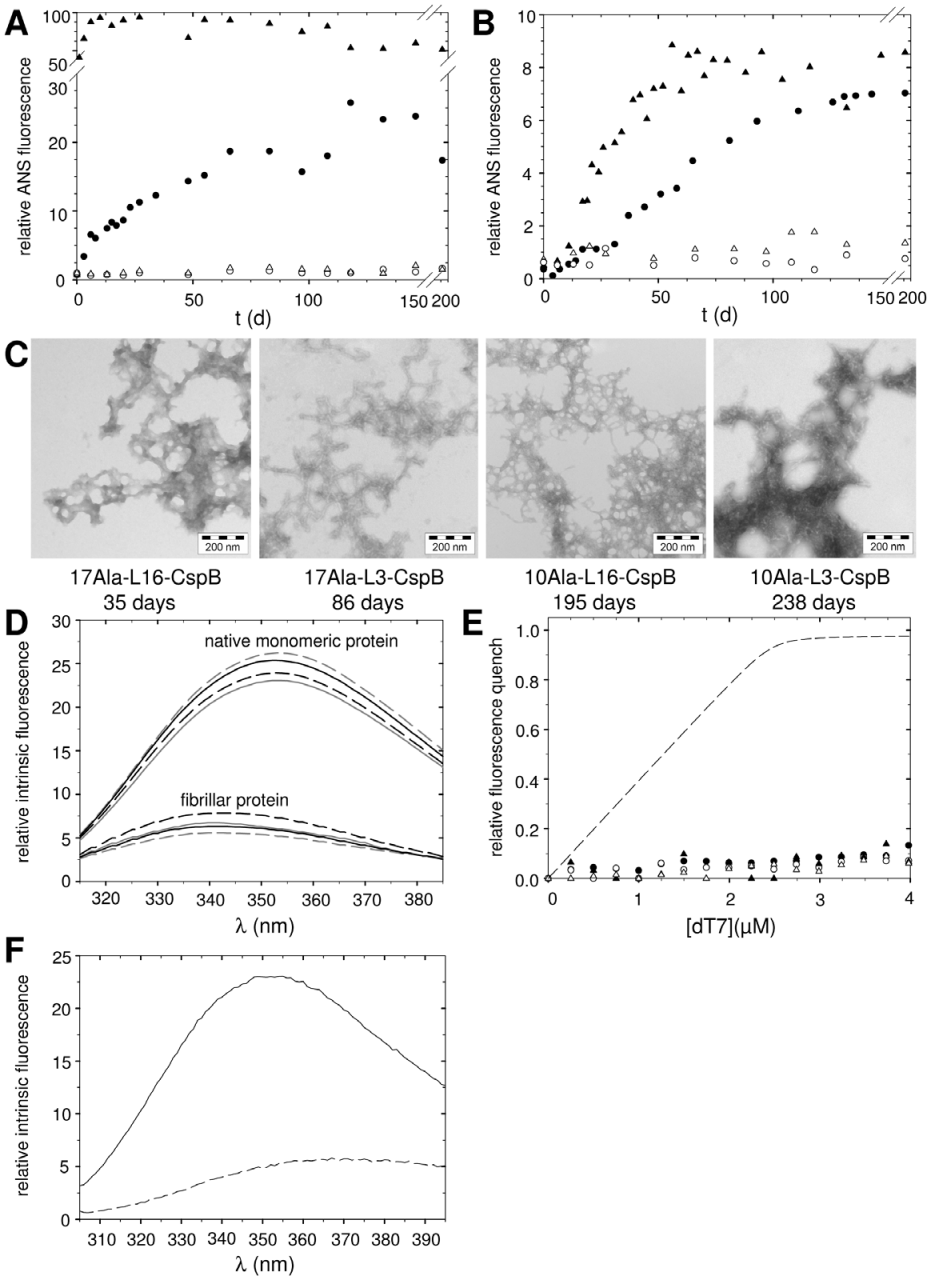
Characterization of the fibrils. The fusions were incubated at a protein concentration of 0.5 mM in 5 mM KH_2_PO_4_, 100 mM NaCl, pH 7.5 at 37°C. **A** ANS fluorescence of the fusion proteins. 17Ala-L16-CspB, filled triangles; 17Ala-L3-CspB, filled circles; 17Ala-L16-MCspB, open triangles; 17Ala-L3-MCspB, open circles. **B** ANS fluorescence of the fusion proteins. 10Ala-L16-CspB, filled triangles; 10Ala-L3-CspB, filled circles; 10Ala-L16-MCspB, open triangles; 10Ala-L3-MCspB, open circles. **C** Electron micrographs of the fusions after incubation times of 35, 86, 195 and 238 days. **D** Fluorescence spectra of the native monomeric fusions and the variants in the fibrillar state. L3-variants are shown in gray; L16-variants in black; variants with 10 alanines are shown as solid line; variants with 17 alanines as dashed line. **E** Binding of dT7 to CspB in the fibrils. Variants with 10 alanines are shown with filled symbols; variants with 17 alanines with open symbols; variants with a linker of 3 amino acids are shown as circles; variants with a linker of 16 amino acids as triangles. The dashed line corresponds to the binding of dT7 to monomeric 10Ala-L3-CspB. The negative controls, variants without alanines (ΔAla-L3/L16-CspB), did not form fibrils over the observed time period. **F** Fluorescence spectra of the protease-digested and solubilized fibrils. 17Ala-L3-CspB, solid line; 17Ala-L16-CspB, dashed line.

Next, the capacity of the fusions to fibrillize was examined. Fibrillation was followed via ANS (8-anilino-1-naphtalene sulfonic acid) or ThT (Thioflavin T) fluorescence and electron microscopy (EM). As shown earlier by us and others for oligo-Ala fibrils, ANS fluorescence changes proved to be more sensitive for monitoring changes upon fibrillization than ThT fluorescence measurements (representative curves for 17Ala-L16-CspB, are shown in the supporting information, Fig. S3) [15], [34]. Thus, ANS fluorescence was used to assess conformational transitions. The variants with 17 Ala showed an immediate increase in ANS fluorescence (Fig. 5A). These increases were always considerably faster when the linker consisted of 16 residues (in 17Ala-L16-CspB) instead of 3 residues as in 17Ala-L3-CspB (Fig. 5A, compare triangles versus circles). Analysis via EM, demonstrated that the increases in ANS fluorescence were not necessarily paralleled by fibril formation, but also indicated amorphous aggregates (data not shown). Clearly discernible fibrillar structures of 17Ala-L16-CspB and 17Ala-L3-CspB were detectable only after incubation times of 35 and 86 days, respectively (Fig. 5C). For the variants with 10 alanines, the ANS fluorescence increased very slowly (Fig. 5B), and significant increases were observed only after 20 and 40 days for 10Ala-L16-CspB and 10Ala-L3-CspB, respectively (Fig. 5B). Here, fibrils could be detected via EM only after approximately 200 days (Fig. 5C). The variants without an oligo-Ala stretch (ΔAla-L3/L16-CspB) neither interacted with ANS nor did they form fibrils (supporting information, Fig. S4).

In the fibrils of the L3 and L16 variants, the fluorescence maximum of Trp8 of the CspB moiety is shifted from 352 nm to 340–343 nm (Fig. 5D), indicating that Trp8 is located in a less polar environment than in isolated CspB, probably because it is incorporated into the fibrils. Similar blue shifts have been reported earlier by us to indicate incorporation of tryptophan residues into fibrillar structures [35]. As part of the fibrillar species, CspB was no longer able to bind to the dT7 oligonucleotide, an observation that confirms that the protein had lost its native structure (Fig. 5E). Taken together, these results show that even a linker of 16 residues between the oligo-Ala stretch and CspB was not sufficient to maintain CspB in a folded conformation within the fibril.

To ascertain whether Trp8 is incorporated into the fibrillar core, fibrils of 17Ala-L3-CspB and 17Ala-L16-CspB were digested with proteinase K. Protease-resistant material was solubilized and analyzed via intrinsic fluorescence spectroscopy. The excitation of Trp8 resulted in the typical tryptophan emission spectrum of monomeric 17Ala-L3-CspB (Fig. 5F, solid line) indicating that the Trp is protected in the fibrillar core. In contrast, with protease-treated fibrils of 17Ala-L16-CspB a typical tryptophan emission spectrum could not be obtained (Fig. 5F, dashed line). Therefore, it can be assumed that Trp8 is not fully integrated in the fibrils of 17Ala-L16-CspB. Still, the fluorescence spectrum of the intact fibrils of 17Ala-L16-CspB (Fig. 5D) indicates that Trp8 is located very close to the fibrillar core.

### Stabilization of CspB inhibits spontaneous and seeded fibril formation

If fibril formation destabilizes the folded conformation of CspB, an increase in the thermodynamic stability of CspB should, in a reciprocal fashion, interfere with fibril formation. To examine this, we constructed fusions (Fig. 1) with a strongly stabilized variant, abbreviated here with MCspB [20]. It contains the two substitutions A46K and S48R, which increase the stability of CspB more than twofold, from 12.4 kJ/mol to 26.9 kJ/mol (Table 1). The folded structure of MCspB and its high affinity for dT7 is not changed in the presence of the N-terminal fusions as shown by far UV-CD spectroscopy and titrations with dT7 (supporting information, Fig. S5A, B). Together, these results demonstrate that, in the soluble fusion proteins, MCspB is folded and functional (Table 1).

The thermodynamic stabilities of the variants with MCspB were derived from urea-unfolding experiments. Almost identical transitions were observed for 10Ala-MCspB and 17Ala-MCspB, as shown for the corresponding L3 and L16 variants in Fig. 4B. Unfolding of all variants was fully reversible, and identical curves were obtained in refolding experiments (supporting information, Fig. S2B).The presence of an N-terminally fused peptide generally decreased the stability of MCspB by about 10 kJ/mol, irrespective of the lengths of the linker and the Ala stretch (Table 1), but MCspB in the fusions was still significantly more stable than isolated wild-type CspB.

For the MCspB fusions, fibril formation could not be observed by ANS binding and electron microscopy. The ANS fluorescence remained low for the variants, in which MCspB was fused to 17 Ala (Fig. 5A, open symbols), even after 200 days of incubation, in contrast to the immediate increases in ANS fluorescence observed for the corresponding variants with wild type CspB (Fig. 5A, filled symbols). The variants with 10 Ala fused to MCspB did not form fibrils either (Fig. 5B, open symbols). When fibril formation was investigated by EM, fibrils could be detected only for fusions containing wild type CspB, but not for those containing MCspB (data not shown).

**Figure 6:**
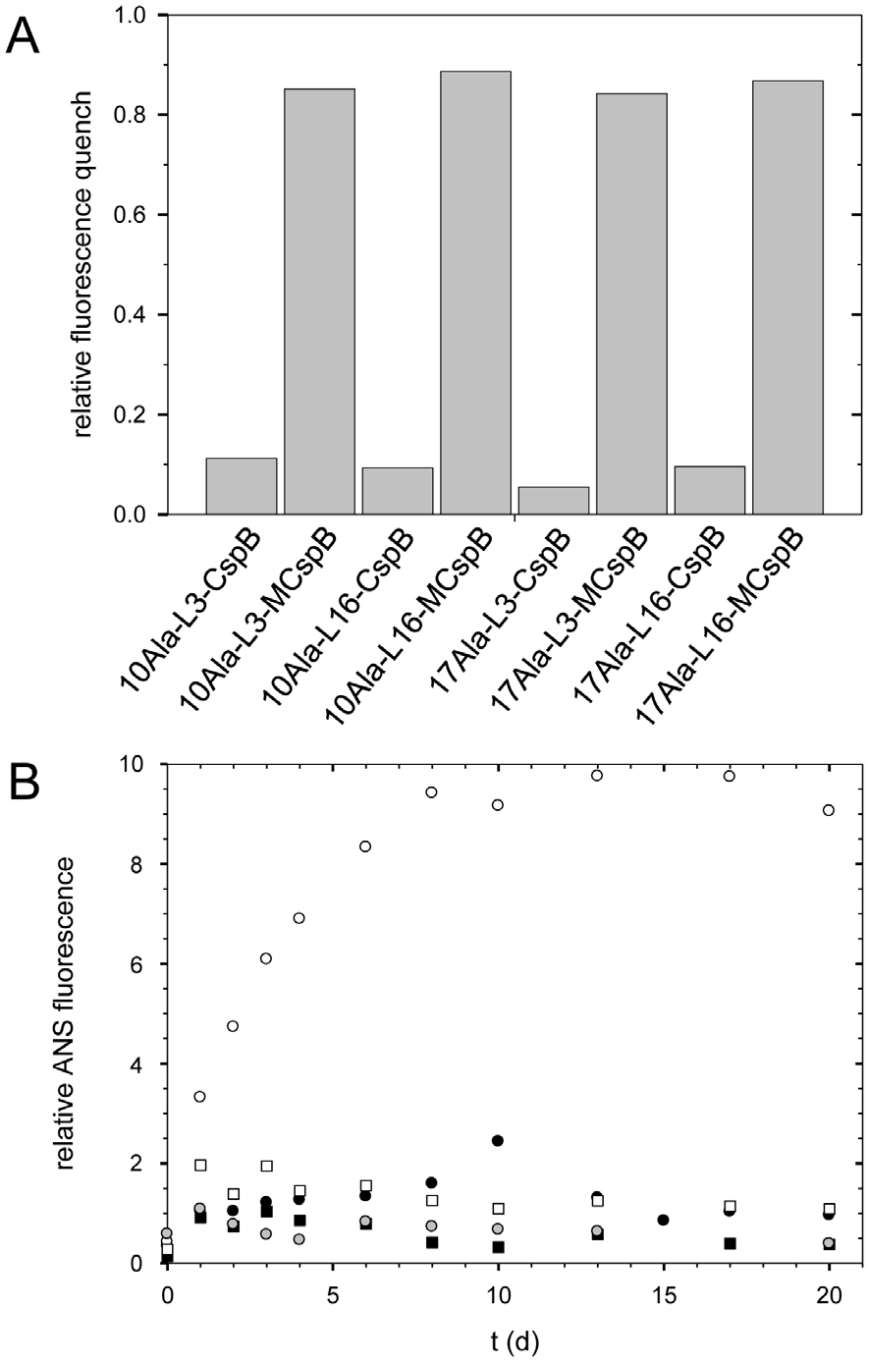
Fibril formation of fusions with MCspB. A Binding of dT7 to CspB in the fibrillation samples after 238 days. **B** Seeding of the variants with 10 alanines. Fibrillations with 5% seeds (w/w) derived from fibrils of 10Ala-CspB as shown with open symbols; fibrillations w/o seeds with filled symbols; 10Ala-L16-CspB (circles); 10Ala-L16-MCspB (squares); 5% seeds alone (gray circles). Fibrillations were performed at a protein concentration of 0.5 mM in KH_2_PO_4_, 100 mM NaCl, pH 7.5 at 37°C.

To examine whether the CspB moiety in the fusions had retained its native structure, all fusion proteins were incubated under fibrillation conditions for more than 200 days. Then their capacity to bind dT7 was assayed. For all fusions with wild type CspB, a fluorescence decrease of only approximately 10% was observed (Fig. 6A), indicating that the capacity to interact with dT7 was largely lost. For the fusions with the stabilized variant MCspB, strong fluorescence decreases of ca. 90% were observed (Fig. 6A), as in the case of the soluble CspB variants. This result indicates that in the fusions with MCspB, the CspB moieties were properly folded and functional, even after the prolonged incubation periods of approximately 200 days at 37°C.

Fibril formation is a nucleation-dependent process and can be accelerated by seeding. In fact, fibril formation of 10Ala-L16-CspB could be accelerated by seeding with fragments of 10Ala-CspB fibrils (Fig. 6B, open circles). Here, the addition of seeds led to an immediate onset of fibril formation, unlike in the control sample without seeds (Fig. 6B, filled circles). For the fusions with MCspB, which failed to fibrillize within 200 days, fibrillation could not be induced by seeds (Fig. 6B, squares). Apparently, the stabilization of CspB renders the fusion protein resistant to fibrillation, even in the presence of seeds.

## Discussion

The amyloidogenic oligo-Ala stretch of the N-terminal domain of the protein PABPN1 forms fibrils that have been discussed to cause OPMD [8]. To examine how fibril formation is affected by the conformational stability of an adjacent folded protein domain, PABPN1 itself could not be used, because the domain following the oligo-Ala stretch cannot be unfolded and refolded reversibly *in vitro* [36]. Instead, we fused CspB in various constructs with the amyloidogenic oligo-Ala stretch of PABPN1. CspB folds fast and reversibly in a simple two-state reaction [18].

Our results indicate that thermodynamic stabilization of a neighbouring folded domain has a drastic effect on the propensity of an amyloidogenic peptide sequence to form fibrils. Unlike in previous investigations of the correlation between protein stability and fibril formation [37], [38], in this work, the independently folding domains were fused to amyloidogenic segments.

The conformational stability of isolated wild-type CspB is low (Δ*G*_D_ = 12.4 kJ/mol), and, at 25°C, about one out of 150 CspB molecules is in the unfolded state. The stability is decreased further when the CspB moiety is fused with amyloidogenic segments. This, together with the high rate of unfolding of CspB [18], probably explains why amyloidogenic sequences such as 10 or 17 Ala stretches as in our fusion proteins are able to pull CspB into β-cross structures. Accordingly, a strong stabilization by mutations, as in MCspB, completely inhibited fibril formation. We refrained from stabilizing the CspB simply by solvent additives, because oligo-Ala induced fibril formation is markedly sensitive to changes in the composition of the solvent [36].

Amyloid fibril formation could occur from unfolded or partially unfolded forms of a protein [39], [40]. Integration of the N-terminal part of CspB into the fibrillar core has been demonstrated by the protease mapping experiments. From a large collection of CspB mutants [41], the CspB double mutant A46K/S48R was chosen because the residues at the positions 46 and 48 in strand β4 improve the stabilizing contacts with the N-terminal strand β1. Thus, this element of secondary structure is much better integrated into the cooperative structure of CspB. The corresponding increase in local stability presumably blocks local unfolding reactions that are necessary to initiate fibril formation.

The mutual interdependence between the conformational stability of the fused protein and the tendency to form fibrils persisted even when the linker between the amyloidogenic sequence and the globular domain was extended from three to 16 residues. The linker length had, however, a significant effect on the lag-phase of fibril formation. The fusions with a linker of 16 amino acids between the alanine residues and wild type CspB formed fibrils always faster than the variants with a linker of three residues. This observation was unexpected because, in the variants with L3 linkers, CspB showed a lower stability than in those with the L16 linkers. Apparently, the rate of fibril formation is determined by both the conformational stability of the folded domain and its distance from the amyloidogenic segment. Yet, we can currently not exclude effects of the linker primary sequence on fibril formation.

The protease-mapping experiments of 10Ala-CspB suggested that only approximately ten N-terminal residues of CspB are incorporated into the core of the fibrils when the CspB moiety was directly linked to the oligo-Ala stretch. Thus, our initial expectation that the extension of the linker to 16 residues would uncouple the CspB moiety from the fibrillar core turned out to be wrong. On the other hand, protease mapping only allows for an approximate assessment of the extent of the fibrillar core, because currently no information is available on the accessibility of either the protease active site or substrate peptides in the probably densely packed peptides close to the fibrillar core. In addition, dense packing resulting in steric hindrance may lead to CspB unfolding in a process that is only indirectly caused by fibril formation.

## Supporting Information

Table S1 **Peptide fragments of the proteinase K digested and solubilized fibrils of 10Ala-CspB.** The highlighted mass indicates the dominant peak of the spectrum. The masses were monoisotopic with [M+H]^+^.
(DOC)

Figure S1 **Coomassie-stained SDS-polyacrylamide gel of the purified fusion variants.**
(TIF)

Figure S2 **Urea-induced refolding transitions of the fusions with CspB and MCspB.** Variants with 10 alanines are shown with filled; variants with 17 alanines with open symbols; variants with linkers of 3 amino acids are shown as circles; variants with linkers of 16 amino acids as triangles. Measurements were carried out in 5 mM KH_2_PO_4_, 100 mM NaCl, pH 7.5 at 20°C. **A** Transitions of the fusions with CspB, **B** Transitions of the fusions with MCspB.
(TIF)

Figure S3 **Comparison of ANS and ThT fluorescence signals of 17Ala-L16-CspB during fibril formation.** Filled symbols correspond to ThT, open symbols to ANS signals.
(TIF)

Figure S4 **ANS fluorescence of fusion proteins without alanines.** The fusions were incubated at concentrations of 0.5 mM in 5 mM KH_2_PO_4_, 100 mM NaCl, pH 7.5 at 37°C. ΔAla-L16-CspB, filled triangles; ΔAla-L3-CspB, filled circles.
(TIF)

Figure S5 **Characterization of the variants with MCspB by far-UV-CD (A) and dT7 binding (B).** In **A**, variants with 10 alanines are black, variants with 17 alanines are gray; variants with the L3 linker, dashed line and with L16 linker, dotted-dashed line. As a reference, the spectrum of MCspB is shown in the black, solid line. In **B**, variants with 10 alanines are shown by filled symbols, variants with 17 alanines by open symbols; variants with L3 linkers are indicated by circles, variants with L16 linkers by triangles. The corresponding K_D_ values are listed in table 1. Measurements were carried out in 5 mM KH_2_PO_4_, 100 mM NaCl, pH 7.5 at 20°C.
(TIF)
