# Supplementary material for: Influence of the Stability of a Fused Protein and Its Distance to the Amyloidogenic Segment on Fibril Formation
Source: PLoS One. 2010 Nov 23;5(11):e15436. doi: 10.1371/journal.pone.0015436 (PMC2990761; doi:10.1371/journal.pone.0015436)
Supplement: Table S1 — Peptide fragments of the proteinase K digested and solubilized fibrils of 10Ala-CspB. The highlighted mass indicates the dominant peak of the spectrum. The masses were monoisotopic with [M+H]+. (DOC) [file pone.0015436.s001.doc]

|  | **mass (Da)** | **theoretical mass (Da)** | **fragment** | **peak** |  |
| --- | --- | --- | --- | --- | --- |
|  |  |  |  |  |  |
|  | 3600.848 | 3600.812 | 4 – 40 | 1 |  |
|  | 3687.898 | 3687.853 | 3 – 40 | 1 |  |
|  | **3716.964** | 3716.912 | 13 – 50 | 2 |  |
|  | 3803.977 | 3803.944 | 12 – 50 | 2 |  |
|  | 3891.025 | 3890.976 | 11 – 50 | 2 |  |
|  |  |  |  |  |  |
