# Supplementary figures and images for: Influence of the Stability of a Fused Protein and Its Distance to the Amyloidogenic Segment on Fibril Formation
Source: PLoS One. 2010 Nov 23;5(11):e15436. doi: 10.1371/journal.pone.0015436 (PMC2990761; doi:10.1371/journal.pone.0015436)

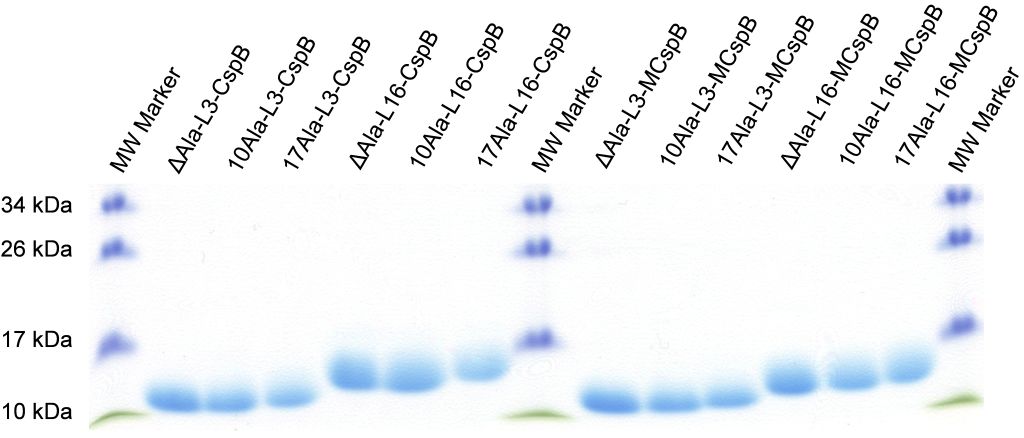

Supplement: Figure S1 — Coomassie-stained SDS-polyacrylamide gel of the purified fusion variants. (TIF) [file pone.0015436.s002.tif]

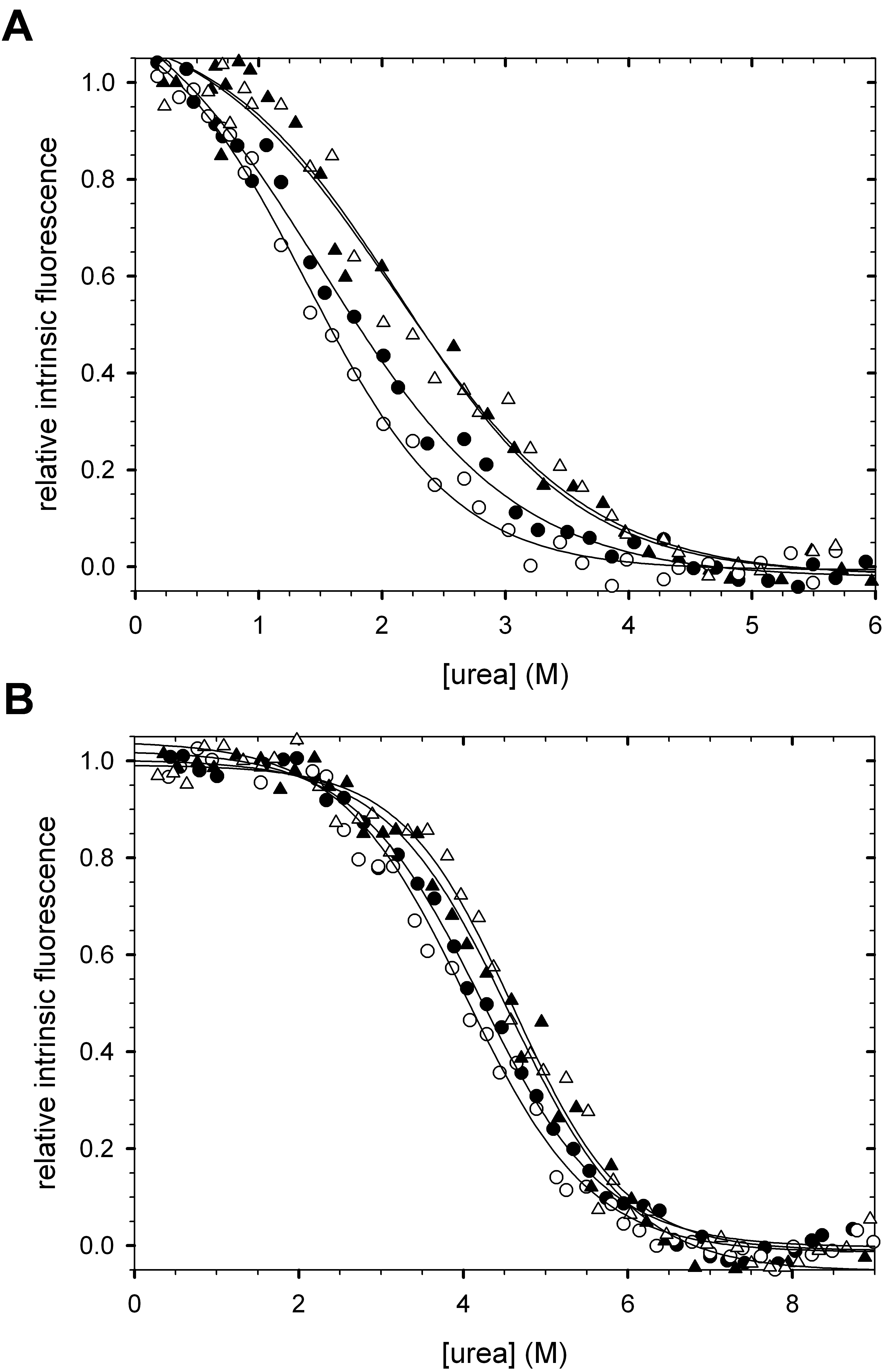

Supplement: Figure S2 — Urea-induced refolding transitions of the fusions with CspB and MCspB. Variants with 10 alanines are shown with filled; variants with 17 alanines with open symbols; variants with linkers of 3 amino acids are shown as circles; variants with linkers of 16 amino acids as triangles. Measurements were carried out in 5 mM KH2PO4, 100 mM NaCl, pH 7.5 at 20°C. A Transitions of the fusions with CspB, B Transitions of the fusions with MCspB. (TIF) [file pone.0015436.s003.tif]

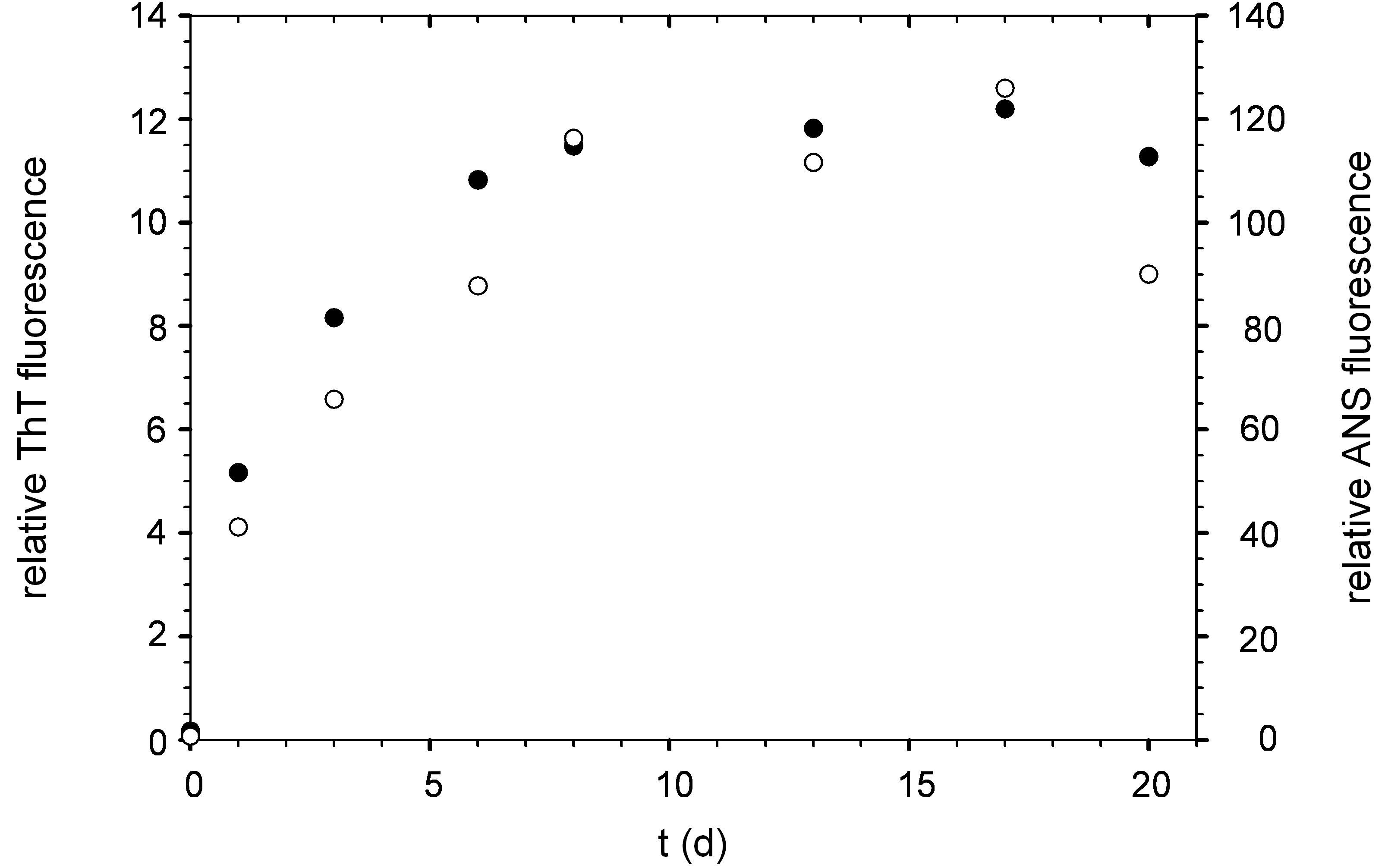

Supplement: Figure S3 — Comparison of ANS and ThT fluorescence signals of 17Ala-L16-CspB during fibril formation. Filled symbols correspond to ThT, open symbols to ANS signals. (TIF) [file pone.0015436.s004.tif]

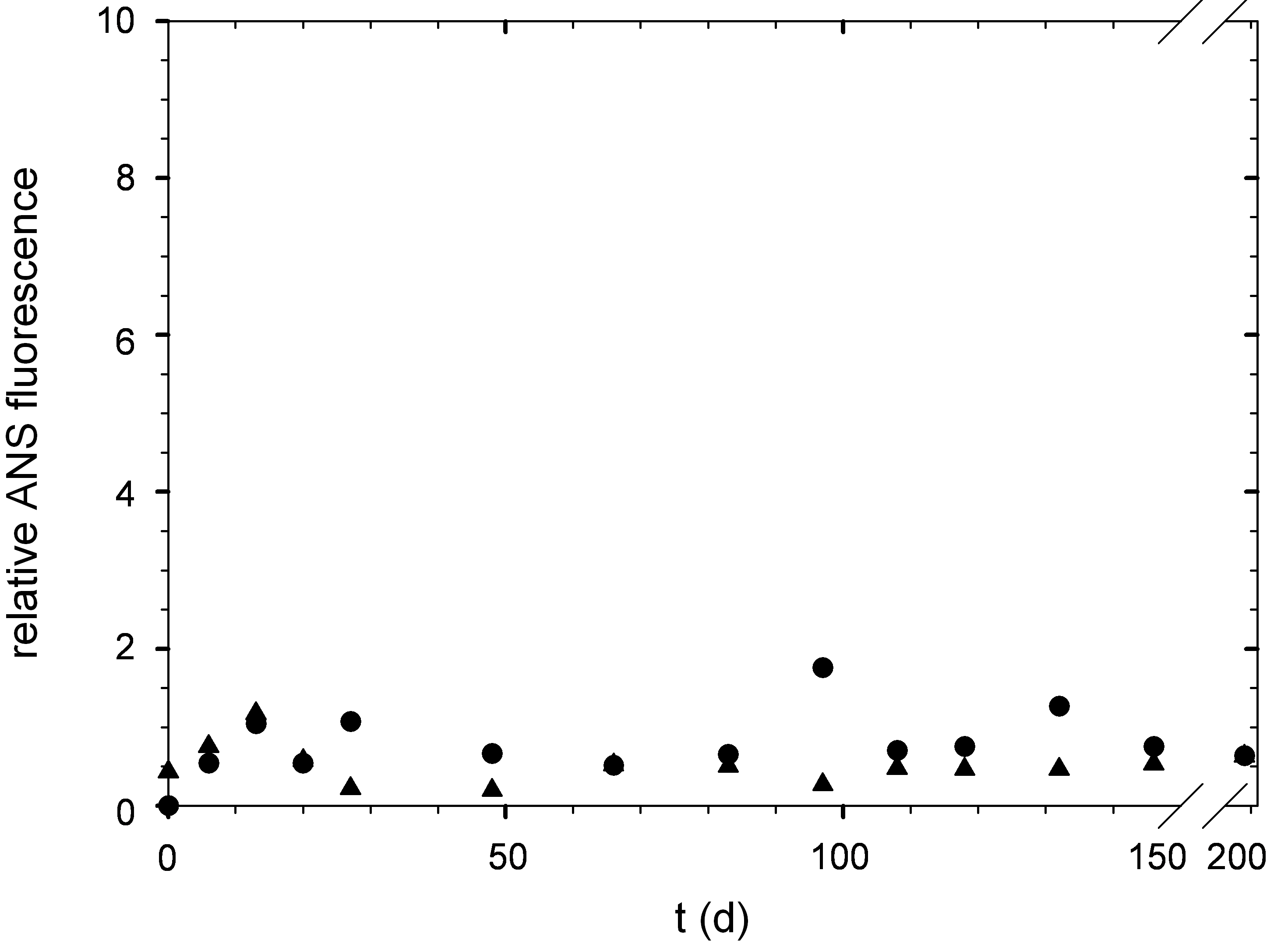

Supplement: Figure S4 — ANS fluorescence of fusion proteins without alanines. The fusions were incubated at concentrations of 0.5 mM in 5 mM KH2PO4, 100 mM NaCl, pH 7.5 at 37°C. ΔAla-L16-CspB, filled triangles; ΔAla-L3-CspB, filled circles. (TIF) [file pone.0015436.s005.tif]

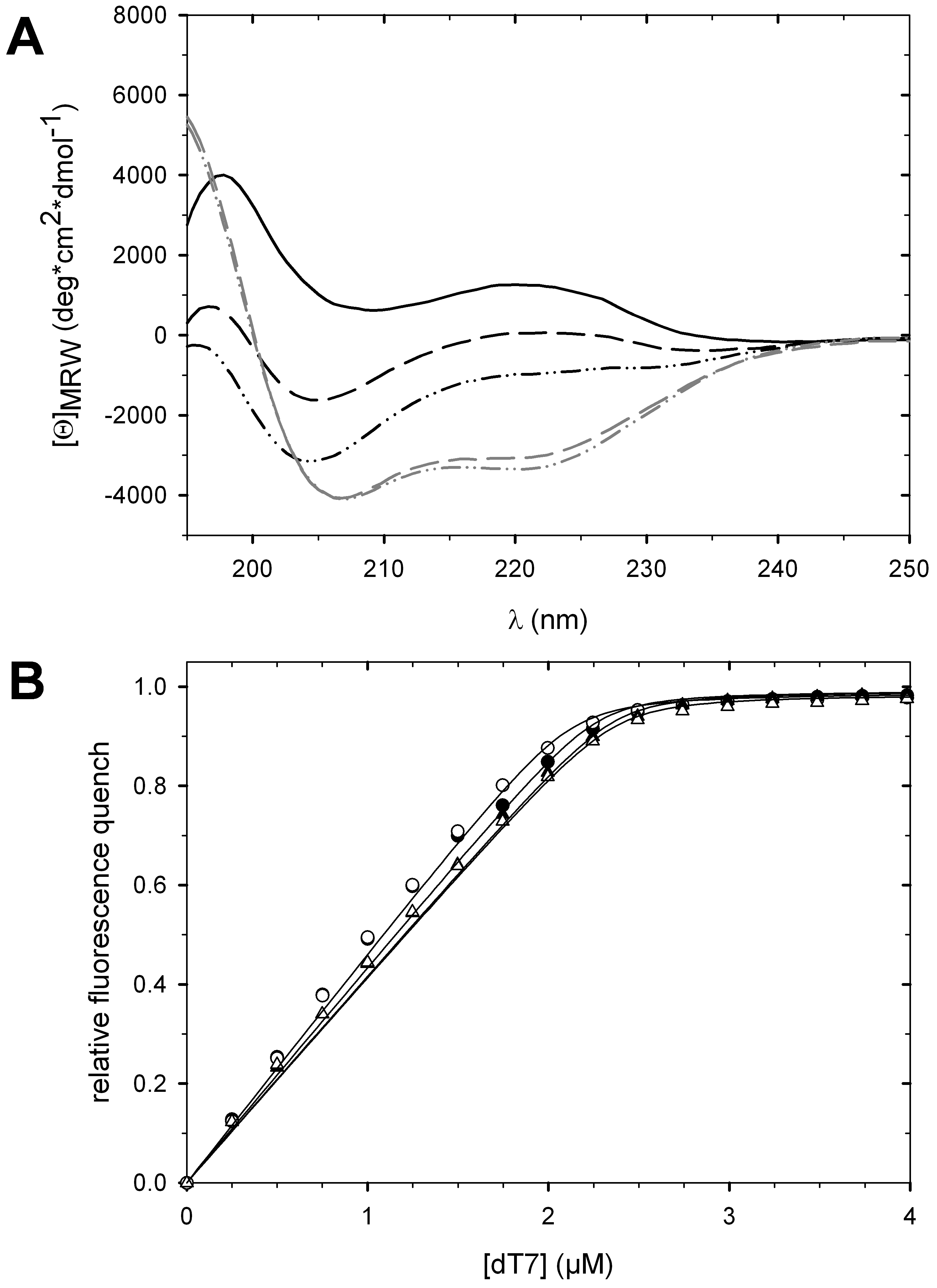

Supplement: Figure S5 — Characterization of the variants with MCspB by far-UV-CD (A) and dT7 binding (B). In A, variants with 10 alanines are black, variants with 17 alanines are gray; variants with the L3 linker, dashed line and with L16 linker, dotted-dashed line. As a reference, the spectrum of MCspB is shown in the black, solid line. In B, variants with 10 alanines are shown by filled symbols, variants with 17 alanines by open symbols; variants with L3 linkers are indicated by circles, variants with L16 linkers by triangles. The corresponding KD values are listed in table 1. Measurements were carried out in 5 mM KH2PO4, 100 mM NaCl, pH 7.5 at 20°C. (TIF) [file pone.0015436.s006.tif]
